# Genome-level identification, gene expression, and comparative analysis of porcine *ß-defensin* genes

**DOI:** 10.1186/1471-2156-13-98

**Published:** 2012-11-15

**Authors:** Min-Kyeung Choi, Minh Thong Le, Dinh Truong Nguyen, Hojun Choi, Won Kim, Jin-Hoi Kim, Jungwan Chun, Jiyeon Hyeon, Kunho Seo, Chankyu Park

**Affiliations:** 1 Department of Animal Biotechnology, Konkuk University, Hwayang-dong,, Kwangjin-gu, Seoul, 143-701, South Korea; 2 College of Veterinary Medicine, Konkuk University, Seoul, South Korea

**Keywords:** Antimicrobial peptide, β-defensins, Pigs, pBD, Pig genome, SNP

## Abstract

**Background:**

Beta-defensins (β-defensins) are innate immune peptides with evolutionary conservation across a wide range of species and has been suggested to play important roles in innate immune reactions against pathogens. However, the complete β-defensin repertoire in the pig has not been fully addressed.

**Result:**

A BLAST analysis was performed against the available pig genomic sequence in the NCBI database to identify β-defensin-related sequences using previously reported β-defensin sequences of pigs, humans, and cattle. The porcine β-defensin gene clusters were mapped to chromosomes 7, 14, 15 and 17. The gene expression analysis of 17 newly annotated porcine β-defensin genes across 15 tissues using semi-quantitative reverse transcription polymerase chain reaction (RT-PCR) showed differences in their tissue distribution, with the kidney and testis having the largest *pBD* expression repertoire. We also analyzed single nucleotide polymorphisms (SNPs) in the mature peptide region of pBD genes from 35 pigs of 7 breeds. We found 8 cSNPs in 7 pBDs.

**Conclusion:**

We identified 29 porcine β-defensin (pBD) gene-like sequences, including 17 unreported pBDs in the porcine genome. Comparative analysis of β-defensin genes in the pig genome with those in human and cattle genomes showed structural conservation of β-defensin syntenic regions among these species.

## Background

Defensins are a large family of cationic cysteine-rich antimicrobial peptides (AMPs) with molecular masses ranging from 2 to 6 kDa; they function as some of the earliest mediators of host defenses in various species of insects, plants, and animals [1-5]. They have a broad spectrum of antimicrobial activity, ranging from bacteria to fungi and some viruses [6]. Defensins are also thought to play a role in connecting innate and adaptive immune responses in higher organisms; they act as signaling molecules in the immune system and chemoattractants for T-lymphocytes and immature dendritic cells [7]. Having both antimicrobial and immunomodulation activity, they are also called “host defence peptides” [8].

Defensins are highly conserved in their structure like defensin fold and function from *Drosophila* to higher mammals [4,6,9-11]. At the sequence level these peptides are remarkably diverse and this appears to have been driven by varying selective pressures and recurrent duplication in mammals [12]. In spite of these interesting features, the functions of most defensins in any organism have not been studied in detail.

On the basis of differences in their size, disulfide bond patterns which are well conserved and related defensin fold, mammalian defensins are classified into α, β, and θ sub-classes [5]. The β-defensins are defined by a 6-cysteine motif, C-X_6_-C-X_4_-C-X_9_-C-X_6_-C-C, where X represents any amino acid residue, and by a large number of basic amino acid residues in their active peptide regions [13,14]. In most cases, their coding sequences consist of 2 exons. The first exon includes the 5′-untranslated region and the leader domain of the preproprotein; the second exon encodes the mature peptide with the 6-cysteine domain [2].

The availability of genomic sequence information has enabled the characterization and comparative analysis of β-defensin repertoires among various species, including humans, chimpanzees, mice, rats, dogs, and chickens [14-18]. Although the role of β-defensins in general immunity against pathogens could be important, limited results are available to elucidate the complete β-defensin repertoire in the pig genome [19,20].

In this study, we characterized 29 functional β-defensin genes in the pig genome on the basis of sequence homology to previously reported β-defensin genes and the conserved 6-cysteine motif. We compared the evolutionary conservation of β-defensin genes among humans, cattle, and pigs, and analyzed their expression patterns. We also report SNPs in the mature peptide region of porcine β-defensin genes.

## Methods

### Identification, annotation, and mapping of porcine β-defensin genes

A BLAST analysis was performed against the high-throughput genome sequences (HTGS) database of *Sus scrofa* at the National Center for Biotechnology Information (NCBI, http://www.ncbi.nlm.nih.gov/) using the previously reported nucleotide sequences of 57 human (DEFB 110, -112, -113, -114, -133, -1, -4, -103, -104, -105, -106, -107, -130, -131, -132, -134, -135, -136, -137, -115, -116, -118, -119, -121, -123, -124, -125, -126, -127, -128, -129, -132), cattle (BBD4, -5, -7, -10, -103A, -103B, -119, -122, -122A, -123, -124,-300, EBD, TAP, LAP), and pigs (pBD1, -2, -3, -4, -104, -108, -114, -123, -125 and -129) β-defensins. Matches with > 70% sequence identity were retrieved and aligned using the ClustalW2 program ( http://www.ebi.ac.uk/Tools/msa/clustalw2/). The exon-intron boundaries were determined by comparing the genomic sequences to available cDNA and EST sequences of human and porcine β-defensins at the NCBI. The GT-AG rule [21] was applied for the prediction of splice junctions when they were not available. The newly described porcine β-defensins were annotated based on nucleotide sequence identity to reported human β-defensins. The nucleotide sequences of identified porcine β-defensins were aligned to the porcine genome assembly (Sscrofa10.2; [22]) using BLAST to determine their positions in the pig genome. The official gene symbols for porcine β-defensins are DEFBs following the assignment of HUGO Gene Nomenclature Committee (HGNC). However, the conventional abbreviation of porcine β-defensins, pBDs, is used here for consistence with previous publications and distinguishing from abbreviations of human β-defensins.

### Phylogenetic analysis

Nucleotide sequences of predicted β-defensin genes were translated in all 6 reading frames using the CLC Main Workbench 5 (CLC bio, Denmark). Amino acid sequences corresponding to correct open reading frames were aligned using ClustalW2 using GONNET Matrix [23] with gap open and extension penalties of 7 and 0.2, respectively. Phylogenetic analyses were performed using MEGA version 5.1 [24]. The evolutionary distances were computed using the JTT matrix-based method [25].

### Preparation of RNA and RT-PCR

Tissues were collected from a 2-week-old and 5-month-old NIH miniature pigs, snap-frozen in liquid nitrogen, and stored at −70°C until use. Total RNA was extracted from small intestine, tongue, eye, cerebrum, spleen, kidney, liver, lung, stomach, testis, muscle, skin, rectum, trachea, and thymus tissues using the RNAiso-Plus^TM^ Reagent (TAKARA, Japan) according to the manufacturer’s instructions. Isolated RNA was subjected to RNase-free-DNaseI treatment (Qiagen, USA) to remove genomic DNA contaminants and was visualized on a 2% formaldehyde agarose gel. Reverse transcription was performed in a 25-μl reaction volume using oligo-(dT)_15_ and SuperScript® III Reverse Transcriptase (Invitrogen, USA) for 50 min at 50°C and inactivated for 15 min at 72°C. For semi-quantitative RT-PCR, 1 μl of the reverse transcription reaction product was used for each tissue in a 15-μl reaction mixture with 10 pmol of each primer (Table 1), 200 μM dNTPs, 0.5 U of SuperTerm® Taq polymerase (LPI, UK), and PCR buffer [10 mM Tris (pH 8.3), 50 mM KCl, and 1.5 mM MgCl_2_]. PCR conditions consisted of 36–42 cycles of 94°C for 30 sec, 56–68°C for 30 sec for primer annealing (Table 1), and 72°C for 30 sec for extension with an initial denaturation step at 94°C for 5 min and a final extension at 72°C for 10 min with a T-3000 thermocycler (Biometra®, Germany). Density values were standardized to glyceraldehyde 3-phosphate dehydrogenase (*GAPDH*) using the primer set: 5′-GCTACACTGAGGACCAGGTTG-3′ and 5′-AGGAGATGCTCGGTGTGTTG-3′. The amplified products were confirmed by sequence analysis to ensure target specificity.

**Table 1:** Polymerase chain reaction (PCR) primers used for the amplification of porcine ß-defensin genes by RT-PCR

** Gene**	**Accession**	**Nucleotides**	**Primer sequences (5′-3′)**	**A.T.**^**b**^	**E.P.**^**c**^
**symbol**	** number**	** position**		**(°C)**	**(bp)**
pBD105	FP102601.2	20573-25396	F - CTCAATTTACATCAGGGTGC	60	135
			R - ACAACCTTCTCGTCCTCAGT		
pBD112	CU041392.3	49146-58607	F - TGTGTAGACGGAAGCTTGAG	60	244
			R - GTCACATTCTCTCATGCAGC		
pBD4^a^		63852-70272	F - GTGGCTTGGATTTGAGGAGAGAGT	58	232
			R - AGTGATACACAGGCCTGGAAGGAT		
pBD114		94921-105728	F - ACCTTGGTGGATCCTGAACGATGC	64	128
			R - TCAAACGCCCTCTGAATGCAGCA		
pBD133		111793-117208	F - GTGCCATGAAAGACACCTAT	60	125
			R - CAGACTTCTCCATGCAACAG		
pBD108^a^	CU442750.3	1195-6843	F - GACGATTGTCATTCTTCTGATCCTGG	58	258
			R - TAGGTTGACTTGTGGTGCCCGAAA		
pBD116		21853-26511	F - CTGATCCTGGTTCATAAGAC	60	211
			R - GAATCCTCCTTCTCGTTAG		
pBD118		75980-87519	F - CTGTTCCTACCACAAGTGAT	60	184
			R - GTGCGAGAAGTGACAGTATT		
pBD119		89342-101888	F - CTGTTTCTTGCCATCCTT	56	168
			R - TACATAGGACTGGAGGCAGC		
pBD122		125631-133938	F - GCTGCACTATTGCTCTTGTC	62	158
			R - TCACACAGCACAGTTTACCA		
pBD123		143565-150992	F - TGGAATCTTCACGGCAAAT	68	100
			R - TGATACTTGGGCTTCACACA		
pBD124		158778-164429	F - CTTCTGCTTATTGTGGCTCT	56	187
			R - ATCTTGGCCATCTTGAGTC		
pBD115	CU627978.3	88572-92708	F - CTTAGCTGTCCTTGTGGTCC	64	227
			R - CAAGCCTTAGCTGTACTTGC		
pBD128		18063-21789	F - GGTTCTCATTATCCTGCTGT	60	258
			R - TGTGTTCACTGTGACAGTGG		
pBD129 ^a^	CU606854.2	119234-124263	F - CAAAGACCACTGTGCCGTGAATGA	58	239
			R - TTGATGCTGGCGAAAGGGTTGGTA		
pBD3 ^a^			F - CTTCCTATCCAGTCTCAGTGTTCTGC	58	308
			R - GGCTTCTGTAGACTTCAAGGAGACAT		
pBD104 ^a^			F - TCCTTCCACGTATGGAGGCTTGTT	58	332
			R - TTACAATACCTCCGGCAGCGAGAA		

^a^[19].

^b^ Annealing temperature.

^c^ Expected product size.

### Cloning and sequencing

PCR products were gel-purified using the QIAquick^TM^ Gel Extraction kit (Qiagen, Germany) and ligated into pGEM-T Easy Vector (Promega, USA). The ligation products were electroporated into DH10B cells (Invitrogen, USA) using a MicroPulser^TM^ (Biorad, USA). Transformed bacteria were plated onto agar containing 50 μg/ml ampicillin, 40 mg/ml X-gal solution, and 100 mM IPTG. The plasmids were isolated using the Plasmid SV Miniprep Kit (GeneAll Biotechnology, Korea). Sequencing reactions were performed using ABI PRISM BigDye^TM^ Terminator 3.1 using T3 and SP6 universal primers. The products were analyzed on an automated DNA Analyzer 3730XL (Applied Biosystem, USA).

### Analysis of nucleotide polymorphisms

Single nucleotide polymorphisms (SNPs) of β-defensin genes were identified from the sequence analysis of the genomic PCR products from 14 animals consisting of 7 breeds, including Landrace, Yorkshire, Berkshire, Duroc, Korean native pigs, Seoul National University (Minnesota) miniature pigs [26,27], and NIH miniature pigs. PCR primers for the amplification of β-defensin exon 2 were designed using primer 3 ( http://primer3.sourceforge.net) ( Additional file 1). The allelic frequency of the identified SNPs was estimated from further genotyping of a total of 35 animals by either PCR-RFLP (Table 2) or sequence analysis of PCR products for identified SNPs.

**Table 2:** Identified nucleotide polymorphisms in the porcine ß-defensin exon 2 region

**Gene**	**SNP position**^**a**^	**Nucleotide**	**Amino acid**^**b**^	**RFLP**	**MAF**^**c**^
pBD1	171	A/G	/	*BstN I*	0.177
pBD4	65	G/A	R/K	*EcoR V*	0.451
pBD113	114	A/G	/	-	0.029
pBD114	186	G/A	/	-	0.09
pBD115	144	A/T	Q/H	-	0.057
pBD115	291	G/A	/	-	0.043
pBD121	96	G/A	/	*Pci I*	0.2
pBD133	196	A/C	K/Q	-	0.043

^a^ The numbering starts from the translation start codon.

^b^ Nonsynonymous changes are indicated by single letter code.

^c^ MAF, minor allele frequency; 35 pigs of 7 breeds (Landrace, Yorkshire, Berkshire, Duroc, Korean Native Pig, Seoul National University miniature Pig, and NIH miniature pig) were used for genotyping.

## Results and discussion

### Identification of 27 porcine β-defensin genes

**Figure 1:**
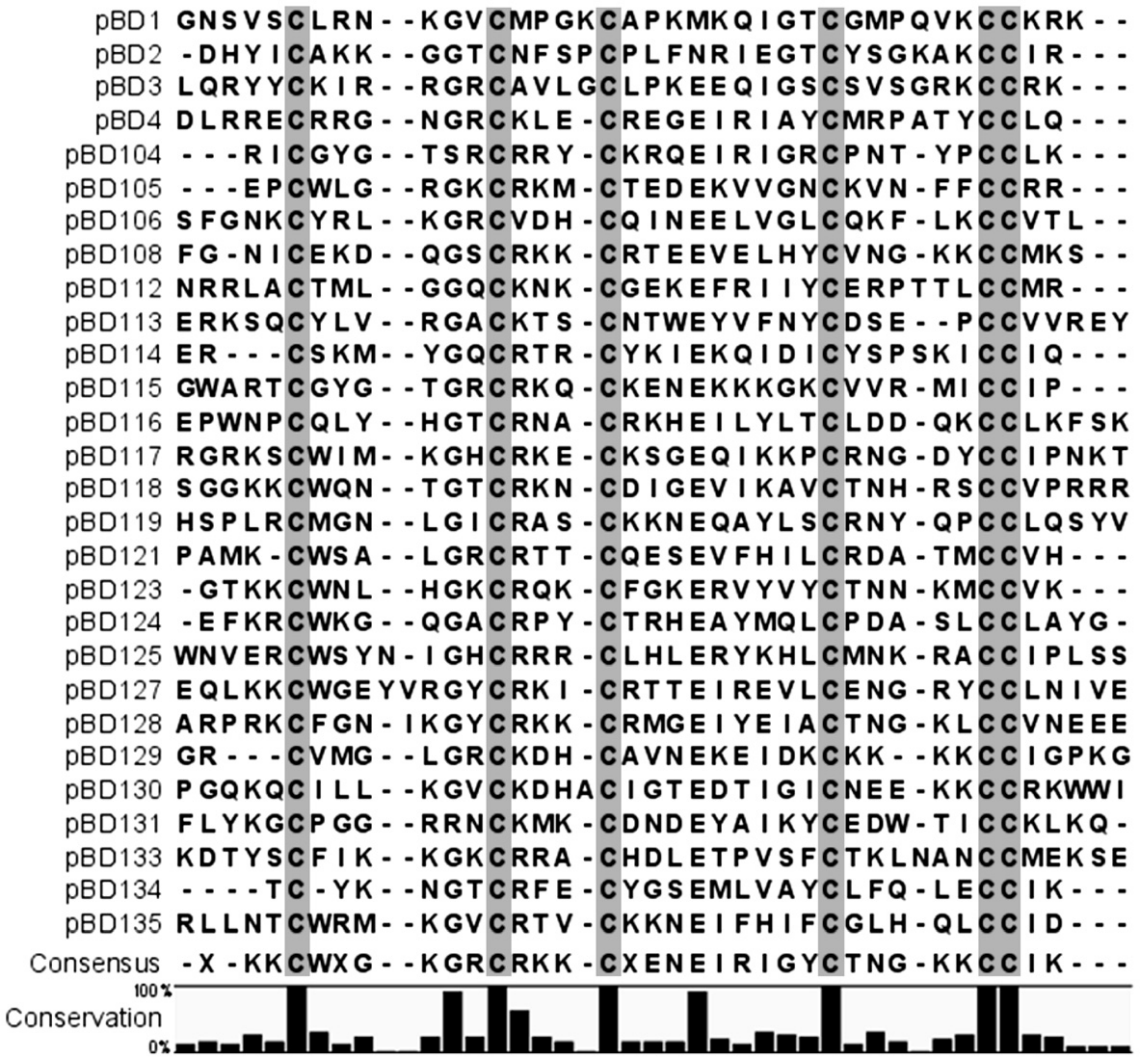
**Comparison of amino acid sequences among 29 porcine β-defensin genes.** Amino acid sequences were predicted from cDNA sequences and aligned using ClustralW2 with minor manual manipulations to maximize sequence alignment. The 6-cysteine motifs are shaded. The 9 significantly conserved sites, including the 6-cysteine motif, are indicated at the bottom.

A BLAST analysis was used to align the pig genomic sequence from NCBI with 57 previously reported β-defensin cDNA sequences from cows, humans, and pigs. We identified 27 matches with >50% sequence coverage and >70% identity to any known β-defensin sequence. In addition, we identified 2 β-defensin genes with relatively lower sequence coverage but higher identity, *pBD112* (32% and 81%, respectively) and *pBD125* (42% and 86%, respectively). The identified putative β-defensin sequences were translated into peptide sequences to determine the open reading frames (ORFs) that contain the 6-cysteine motif, a major characteristic of β-defensins peptides. As a result, we determined 29 ORFs satisfying our criteria for porcine β-defensins, including 17 previously unreported genes (*pBD105, -106, -112, -113, -115, -116, -118, -119, -122, -123, -124, -128, -130, -131, -133, -134 and -135*), 10 reported genes (*pBD1, -2, -3, -4, -104, -108, -114, -121, -125 and -129*) and 2 partial genes (*pBD117****ψ*** and *-127****ψ***) that lacked sequences corresponding to the exon 1 region ( Additional file 2). It was difficult to determine with confidence whether the lack of exon 1 region from *pBD117****ψ*** and *-127****ψ*** was due to a deletion in the pig genome or to an incomplete pig genomic sequence. However, RT-PCR results using specific primers for *pBD117****ψ*** and *-127****ψ*** did not show any evidence of mRNA expression, supporting that these genes are nonfunctional (data not shown). All porcine β-defensins contained abundant positively-charged amino acid residues, such as lysine (K) and arginine (R) (Figure 1).

One way to identify defensins from genome sequence information is to use gene prediction algorithms like the HMM (Hidden Markov Model) that incorporate homology profiling [14,15,17,28]. Although these methods are accurate and easy to use, they usually do not support the identification of a complete list of defensin genes because of inadequate accommodation of the sequence diversity of β-defensins in the sequence homology profile. Therefore, we chose the manual analysis method using NCBI blast analysis.

### Comparative study of porcine β-defensin genes using phylogenetic analysis

**Figure 2:**
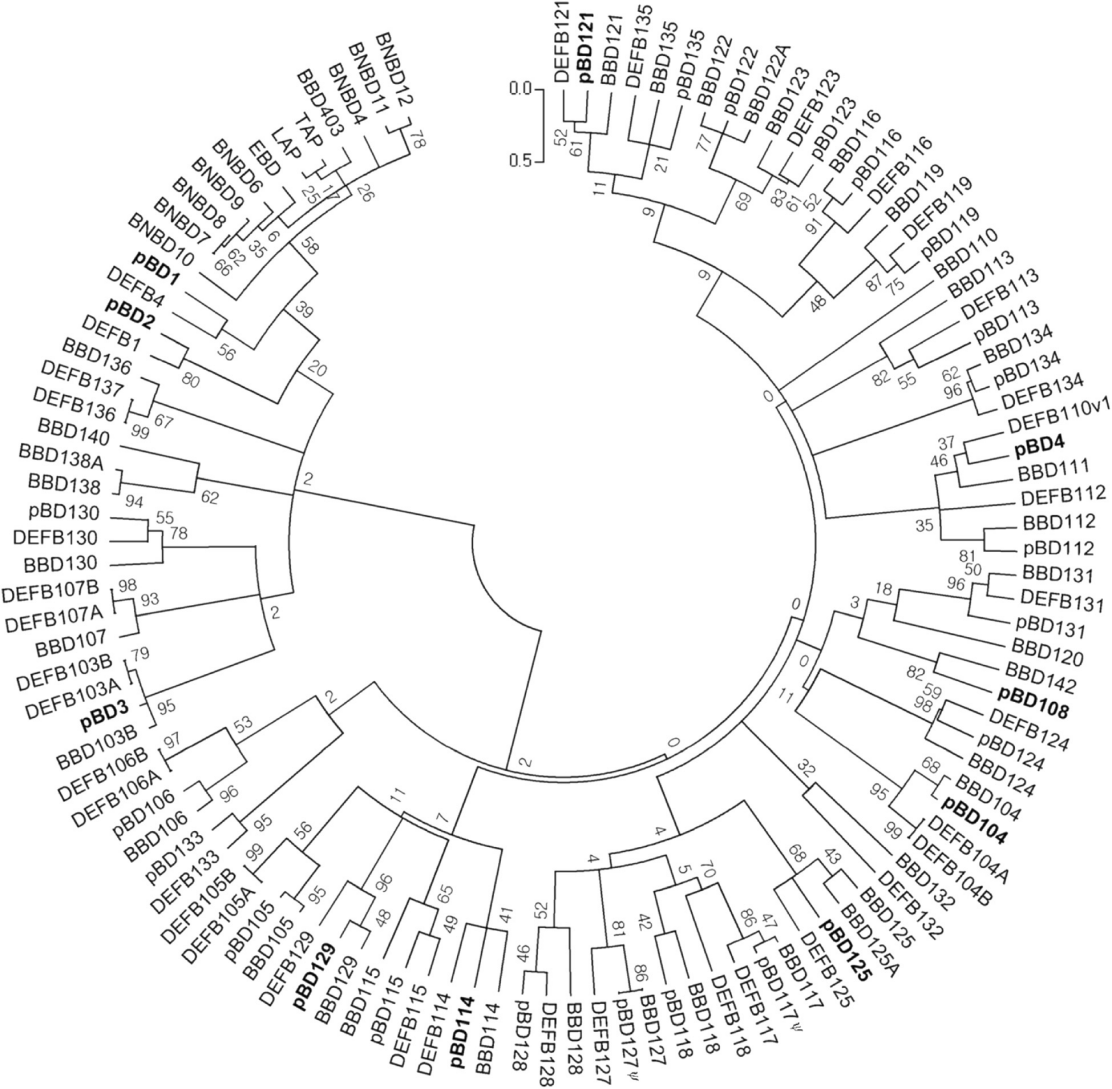
**A phylogenetic analysis of β-defensin genes among humans, cattle, and pigs.** 36 β-defensin genes from humans, 48 from cattle, and 29 from pigs were analyzed using the neighbor-Joining method. The bootstrap consensus tree inferred from 1,000 replicates and branches corresponding to less than 50 % bootstrap replicates were collapsed. The analysis involved 113 amino acid sequences of β-defensin prepropeptides. pBD, β-defensins; BBD, bovine β-defensins; DEFB, human β-defensins. The “*ψ*” symbol of *pBD117ψ* and *-127ψ* indicates Pseudogenes or partial genes. Sequences of human and cattle β-defensin genes [47] were obtained from NCBI.

To annotate the putative β-defensin-encoding sequences identified from our analysis, a phylogenetic analysis was performed using 113 amino acid sequences corresponding to the β-defensin prepropeptide, including signal and mature peptide regions, together with previously reported β-defensins from humans and cattle (Figure 2). We annotated porcine β-defensin genes on the basis of sequence similarity and phylogenetic relationships to previously described β-defensins in humans to maintain consistency in the comparative analysis of β-defensins with other species. The results showed that the nomenclature of 10 previously reported porcine β-defensin genes were consistent with that of human, except for *pBD1–4* and *pBD-123. pBD1, -2, -3,* and -*4*, which were more closely related to *DEFB4, -1, -103*, and -*110* in humans, respectively. Since several studies have investigated *pBD1–4*[19,29-33], it could cause confusion if they were renamed; accordingly, we have maintained their names. However, we suggest renaming previously reported *pBD123*[19] to *pBD121*, considering its closer sequence similarity and phylogenetic relationship to human *DEFB121* than *DEFB123*. This change would make the nomenclature of porcine β-defensins consistent with that of other species. As a result, the orthologs of β-defensin123 from humans, pigs, and cattle become clustered together (Figure 2).

Although it was difficult to define orthologous relationships for some of the β-defensin genes, interspecies sequence identity between presumptive orthologous β-defensins with the same numbers in their names was higher in most cases than the values between non-orthologus β-defensins within the same species ( Additional file 3). The average nucleotide sequence identity from the 27 pairs of orthologous β-defensins between humans and pigs was 84.38%.

*DEFB105* in human consists of 3 exons, in contrast to the typical 2-exon structure of other β-defensin genes [2]. A 1,249 bp nucleotide insertion in exon 2 changed the single exon to 2 exons in *DEFB105*[28]. The porcine orthologous gene, *pBD105*, also consists of 3 exons in the same configuration, suggesting that the insertion event occurred in the common ancestor of humans and pigs. This gene was missing in the current bovine genome assembly [34].

### Localization of porcine β-defensin genes to chromosomes 7, 14, 15, and 17

**Figure 3:**
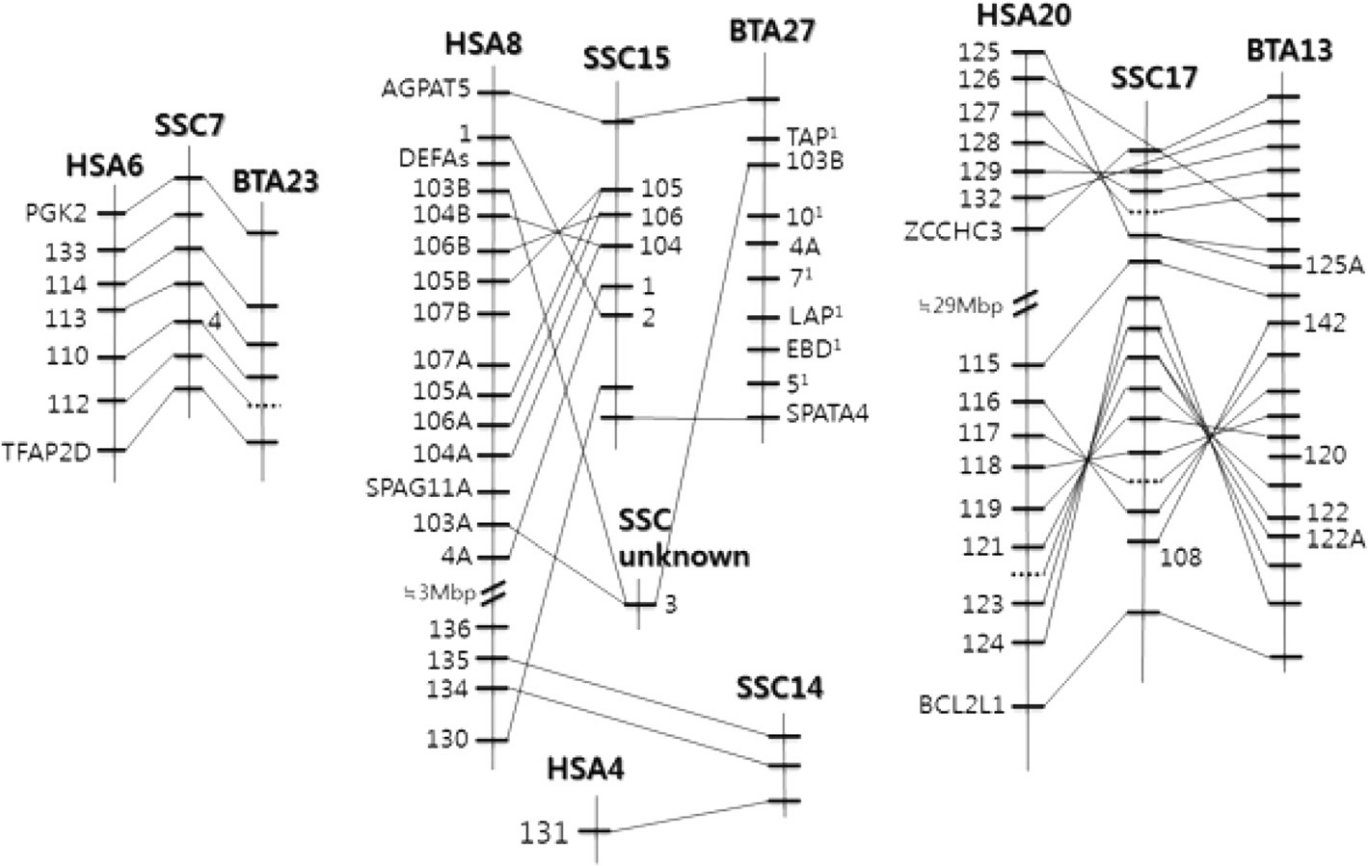
**Comparison of β-defensin-containing chromosomal regions among human, pig, and cattle genomes.** The evolutionarily conserved flanking markers and the clustered β-defensin genes are shown. The genes with orthologous relationships are indicated by lines among maps of different species. The names of the β-defensin genes are indicated with only numbers without species-specific symbols (DEFB for human, BBD for cattle, and pBD for pigs). Pseudogenes or partial genes identified in the pig genome sequencing results at NCBI are shown as dotted lines. Information from genome build 37.2, Sscrofa10.2, and Btau5.2 were used for humans, pigs, and cattle, respectively. ^1^Some of the cattle β-defensin genes have less typical names, including *TAP, LAP,* and *EBD*.

The identified 29 porcine β-defensin related sequences were mapped to the pig genome assembly (Sscrofa10.2) using BLAST to determine their location. They were localized to 4 clusters on 4 pig chromosomes, *Sus scrofa* chromosome (SSC) 7, SSC14, SSC15, and SSC17, with several genes in each cluster (Figure 3). By comparing the available gene annotations for humans and cattle at NCBI with our mapping results on porcine β-defensin genes, we identified the β-defensin-containing syntenic regions for the 3 species with the help of evolutionarily conserved flanking markers around the β-defensin gene clusters, such as *PGK2* and *TFAP2D* for the SSC7 cluster, *pBD135* and −*131* for SSC14, *AGPAT5* and *SPATA4* for SSC15, and *ZCCHC3 (LOC100519451)* and *BCL2L1* for SSC17. For the SSC14 cluster, we directly used the β-defensin genes as evolutionarily conserved markers, since the determination of evolutionarily conserved markers among humans, pigs, and cattle was less clear. Although we further analyzed sequences within these flanking markers for the possible presence of β-defensin-like sequence in the pig genome, no other sequences were found, consistent with the high sequence homology among β-defensin genes ( Additional file 3).

Using information from previous studies [19,34,35] and from the NCBI Map Viewer ( http://www.ncbi.nlm.nih.gov/mapview/), we constructed a comparative map of the syntenic regions of β-defensins among humans, cattle, and pigs (Figure 3). The comparison of the β-defensin syntenic regions among the 3 species showed significant interspecies conservation, including gene orders in the regions, with slight variations specific to each species, supporting the consistency of our annotation of the porcine β-defensin genes. The SSC7 cluster, consisting *of pBD133, -114, -113, -4,* and -*112*, was the most conserved region among the clusters. The SSC15 cluster between AGPAT5 and *SPATA4* contains 6 β-defensin genes*, pBD105, -106, -104, -1, -2*, and -*130*, and the genetic variation within the cluster among cattle, humans, and pigs was somewhat greater than the other regions. For example, the *Homo sapiens* autosome (HSA) 8 cluster was separated into 2 chromosomes, SSC15 (*pBD105, -106, 104, -1, -2,* and -*130*) and SSC14 (*pBD135, -134*, and -*131*), in the pig genome. Also, there were gene duplications in the human cluster compared to those of cattle and pigs. Cattle β-defensin genes in the region showed higher sequence variations compared to homologous regions in humans and pigs; thus, the establishment of orthologous relationships with β-defensin genes among humans, pigs, and cattle was not clear for this region. The largest number of β-defensin genes was found in the SSC17 cluster between *TRIB3* and *BCL2L1* and contained 12 genes, *pBD129, -128, -127, -115, -124, -123, -122, -121, -119, -118, -117,* and -*116*, which were separated into 2 sub-clusters in HAS20. In the current Sscrofa10.2 assembly, the chromosomal location of a linked β-defensin gene, *pBD3*, was not determined although this gene is in contig *NW_003613575.1*. Considering the positions of the orthologous genes in the human genome, the most likely position of *pBD3* in the pig genome is SSC15 (Figure 3).

Absence of α-defensins in the bovine genome was reported previously [36]. Consist to this, there were no α-defensins in the pig genome, suggesting that the α-defensins may not present in the artiodactyla lineage.

### Gene expression analysis of 17 newly annotated porcine ß-defensin genes

We analyzed the expression pattern of 22 β-defensin genes including 17 newly annotated β-defensin genes together with the 5 previously described genes. To evaluate the patterns of β-defensin expression in pigs, we used respiratory (lung and trachea), digestive (tongue, stomach, small intestine, and rectum), reproductive (testis), primary immune (spleen and thymus), and other (eye, cerebrum, kidney, liver, muscle, and skin) tissues. RT-PCRs were designed to distinguish amplicons between genomic DNA and cDNA templates according to their product size (data not shown). To detect the expression of β-defensin genes on an agarose gel, our RT-PCR profiles consisted of 36 to 42 cycles, which is more than typical semi-quanitative PCR, suggesting that the expression level of β-defensins is relatively low in healthy pigs.

**Figure 4:**
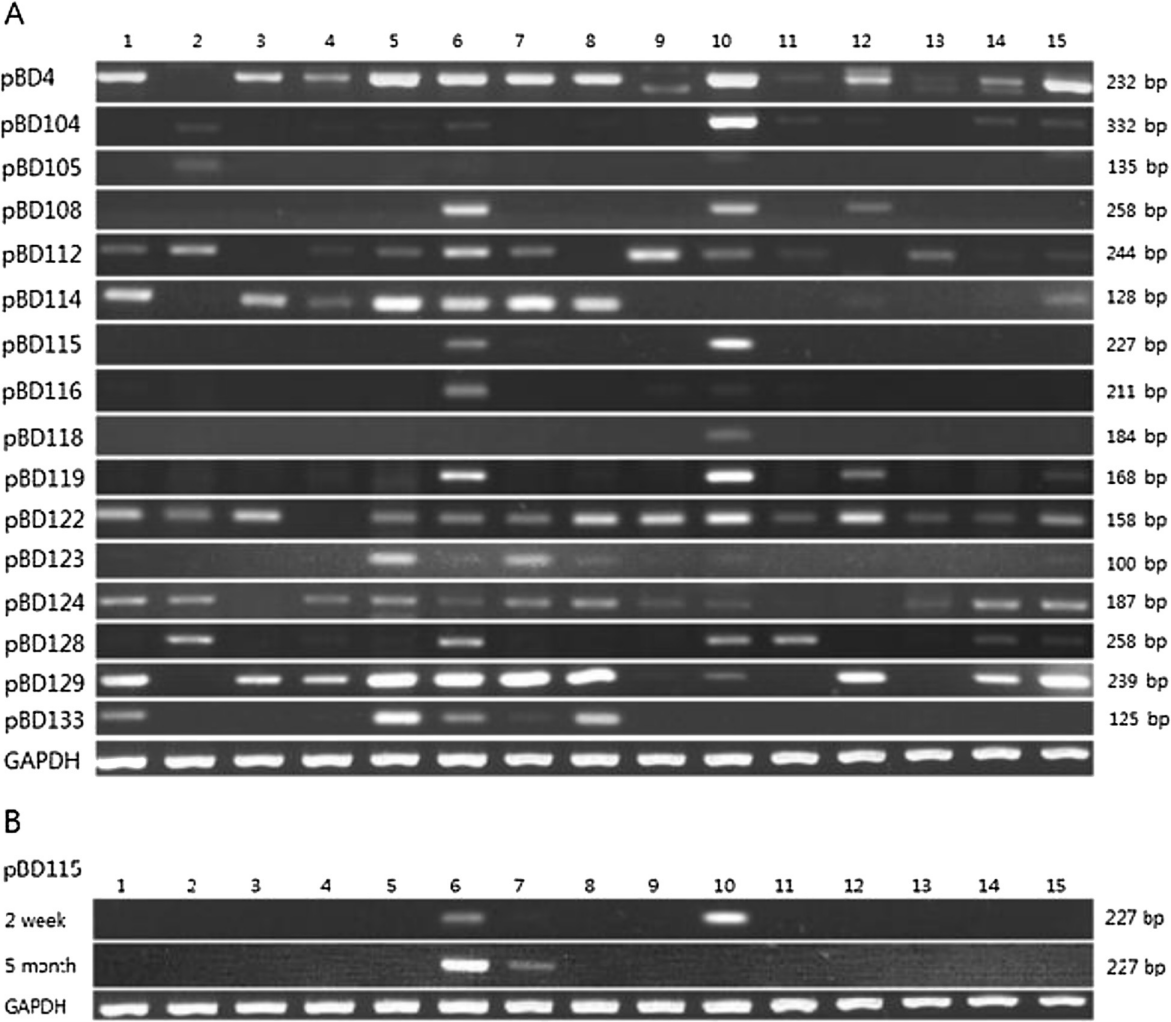
**Analysis of the tissue expression profiles of 16 porcine β-defensins from 15 tissues using semi-quantitative RT-PCR.***GAPDH* was used as a control to control for the amount of cDNA and the level of gene expression. (**A**) The gene expression analysis of β-defensins from a 2-week-old pig. (**B**) Temporal changes in the gene expression of pBD115 in testes between 2-week-old and 5-month-old pigs. 1, small intestine; 2, tongue; 3, eye; 4, cerebrum; 5, spleen; 6, kidney; 7, liver; 8, lung; 9, stomach; 10, testis; 11, muscle; 12, skin; 13, rectum; 14, trachea; 15, thymus.

In our tissue panel, semi-quantitative RT-PCR of the 17 newly annotated β-defensins showed detectable amounts of only 11 genes (*pBD105, -112, -115, -116, -118, -119, -122, -123, -124, -128,* and −*133*) (Figure 4). The other 6 genes (*pBD106, -113, -130 -131, -134,* and -*135*) did not show evidence of mRNA expression from the RT-PCR. Subsequent genomic PCR for these unexpressed genes successfully yielded genomic DNA-specific amplicons (data not shown), suggesting that the amplification failure was indeed due to a lack of expression. Because these non-expressed β-defensins have intact ORFs for the coding regions, further studies are necessary to evaluate the functional importance of these genes, including the induction of gene expression in animals by microbial challenge or analysis in tissues that were not evaluated in this study such as the bone marrow, an immune regulatory organ [37].

The expression patterns of porcine β-defensin mRNAs were consistent with the expected function of β-defensins as antimicrobial peptides. A greater diversity of β-defensins was expressed from the tissues that require strong mucosal defenses, such as the small intestine and lung, and that control the immune system, such as the spleen and thymus (Figure 4). Among porcine the β-defensins, *pBD4, -122*, and -*129* showed strong expression in most pig tissues. The results of our gene expression analysis on 5 previously reported porcine β-defensins genes (*pBD4, -104, -108, -114*, and -*129*) were consistent with those of a previous study [19] with only minor differences in the tissue panel.

Interestingly, the kidney and testis expressed the most diverse β-defensins. It has been suggested that β-defensins control the development of the reproductive system [38-40]. Our analysis on the temporal expression of *pBD115* showed strong expression in the 2-week-old testis but no expression at 5 months (Figure 4B). Other pig β-defensins including *pBD108, -116, -118, -119, -122, -123* and *124* in the syntenic region did not show significant differences in their expression pattern between the two different stages (data not shown). The expression pattern in the kidney was opposite to that of the testis, suggesting that expression may be developmentally regulated.

The presence of porcine β-defensin genes within 4 small clusters on 4 chromosomes allowed us to evaluate possible co-regulation of genes closely located within a cluster. However, adjacent *pBD114* and -*133* showed completely different expression patterns, and pBD4 and -129, on different chromosomes, showed a similar expression pattern (Figures 3 and 4). This suggests that the expression of each β-defensin is independently regulated, even for β-defensins closely located within a cluster.

### Identification of single nucleotide polymorphisms

Many studies have suggested possible associations between SNPs within β-defensin genes and disease susceptibility [41-45]. To identify cSNPs present in the mature peptide-coding region of porcine β-defensins, we evaluated SNPs in exon 2 region of 20 (*pBD1, -2, -4, -104, -105, -108, -112, -114, -115, -116, -118, -119, -121, -122, -123, -124, -125, -128, -129 and -133*) porcine β-defensin genes. In doing so, we identified 8 cSNPs from 7 genes. We found 3 nonsynonymous varionts from pBD4, -115 and -133. We searched for restriction enzymes to perform PCR-RFLP on the identified SNPs. Polymorphisms of *pBD1*, *-4*, and -*121* were distinguishable using *Bst*NI, *Eco*RV, and *Pci*I, respectively. Genotyping was performed for each SNP, and allelic frequencies were estimated (Table 2). It will be interesting to evaluate the possible association between these polymorphisms and innate immunity against pathogens important in pig production.

## Conclusions

AMPs are among the most ancient components of the immune system [46], but their extensive role in mammalian defenses [2] and their positive selection throughout evolution [28] have only recently become apparent. We identified 29 porcine β-defensin (pBD) gene-like sequences, including 17 unreported pBDs in the porcine genome. Although the genome-level characterization of porcine β-defensin genes has demonstrated the existence of multiple genes encoding peptides with possible antimicrobial function, further studies will be required to identify their functional differences or specificity. A better understanding of the roles of porcine β-defensin genes could be useful for improving general health or resistance to microbial infections in pigs.

## Abbreviations

AMP: Anti-microbial peptide; BBD: Bovine β-defensin; BTA: *Bos taurs* autosome; EST: Expressed sequence tag; DEFB: Human β-defensin; GAPDH: Glyceraldehyde 3-phospate dehydrogenase; HAS: *Homo sapiens* autosome; HMM: Hidden Markov model; HTGS: High-throughput genome sequences; pBD: Porcine β-defensin; RT-PCR: Reverse transcription polymerase chain reaction; SNPs: Single nucleotide polymorphisms; SSC: *Sus scrofa* chromosome.

## Competing interests

The authors declare that they have no competing interests.

## Authors’ contributions

MKC was responsible for *in silico* analysis, comparative mapping and phylogenetic analysis of pBDs. MTL and DTN performed the gene expression analysis of pBDs. HC and WK collected pig samples and perform SNP analysis of pBDs. JC and JH worked on cloning of pBDs and sequence analysis. JHK and KS provided helpful ideas and discussion for the experiment. CP was involved in project planning, discussion and writing of the manuscript as a project principle investigator. All authors read and approved the final manuscript.

## Supplementary Material

Additional file 1 Primer sequences used for the analysis of porcine β-defensin exon 2 polymorphisms. Click here for file

Additional file 2 Characterization of 29 porcine β-defensin genes for their exon/intron junctions and coding peptides. Click here for file

Additional file 3 **Analysis of nucleotide sequence identity of β-defensin prepropeptides among humans, pigs, and cattle (see the separate file).** The numbers indicate the value for the pairwise sequence identity. The degree of sequence identity was represented with color gradients from red (high homology), pink, white, to blue (low homology). The most similar sequences were found along the diagonal direction of the table from the top left to the lower right. Click here for file
