# Supplementary material for: Genome-level identification, gene expression, and comparative analysis of porcine ß-defensin genes
Source: BMC Genet. 2012 Nov 15;13:98. doi: 10.1186/1471-2156-13-98 (PMC3499285; doi:10.1186/1471-2156-13-98)
Supplement: Additional file 3 — Analysis of nucleotide sequence identity of β-defensin prepropeptides among humans, pigs, and cattle (see the separate file). The numbers indicate the value for the pairwise sequence identity. The degree of sequence identity was represented with color gradients from red (high homology), pink, white, to blue (low homology). The most similar sequences were found along the diagonal direction of the table from the top left to the lower right. [file 1471-2156-13-98-S3.pdf]

		1	2	3	4	5	6	7	8	9	10	11	12	13	14
DEFB110v1	1		76.12	70.15	45.59	19.35	16.67	23.53	23.29	26.03	23.29	23.29	18.06	16.67	13.08
pBD4	2	76.12		74.63	45.59	19.35	16.67	24.71	26.03	27.40	27.40	27.40	22.22	20.83	15.89
BBD111	3	70.15	74.63		48.53	20.43	17.54	24.71	26.03	28.77	27.40	27.40	19.44	19.44	14.95
BBD110	4	45.59	45.59	48.53		22.34	18.26	26.74	20.83	25.00	23.61	23.61	21.43	21.43	17.14
BBD113	5	19.35	19.35	20.43	22.34		53.64	47.78	11.34	13.40	12.37	12.37	11.34	10.31	11.71
pBD113	6	16.67	16.67	17.54	18.26	53.64		40.54	9.48	12.07	11.21	11.21	10.34	9.48	11.54
DEFB113	7	23.53	24.71	24.71	26.74	47.78	40.54		12.36	15.73	14.61	14.61	15.73	14.61	15.89
BBD103B	8	23.29	26.03	26.03	20.83	11.34	9.48	12.36		80.60	74.63	74.63	40.30	40.30	27.45
pBD3	9	26.03	27.40	28.77	25.00	13.40	12.07	15.73	80.60		79.10	79.10	37.31	37.31	26.47
DEFB103A	10	23.29	27.40	27.40	23.61	12.37	11.21	14.61	74.63	79.10		100.00	43.28	41.79	28.43
DEFB103B	11	23.29	27.40	27.40	23.61	12.37	11.21	14.61	74.63	79.10	100.00		43.28	41.79	28.43
BNBD11	12	18.06	22.22	19.44	21.43	11.34	10.34	15.73	40.30	37.31	43.28	43.28		95.00	53.54
BNBD12	13	16.67	20.83	19.44	21.43	10.31	9.48	14.61	40.30	37.31	41.79	41.79	95.00		50.51
BBD403	14	13.08	15.89	14.95	17.14	11.71	11.54	15.89	27.45	26.47	28.43	28.43	53.54	50.51	
EAP	15	20.55	21.92	20.55	22.86	15.46	13.79	21.35	39.71	39.71	42.65	42.65	73.85	69.23	55.56
BNBD7	16	20.83	22.22	20.83	23.53	13.40	12.07	17.98	39.71	39.71	39.71	39.71	65.62	60.94	49.49
[illegible]															
